# Effects of Spatial Localization on Microbial Consortia Growth

**DOI:** 10.1371/journal.pone.0168592

**Published:** 2017-01-03

**Authors:** Michael Venters, Ross P. Carlson, Tomas Gedeon, Jeffrey J. Heys

**Affiliations:** 1 Chemical and Biological Engineering Department, Montana State University, Bozeman, Montana, United States of America; 2 Department of Mathematical Sciences, Montana State University, Bozeman, Montana, United States of America; Purdue University, UNITED STATES

## Abstract

Microbial consortia are commonly observed in natural and synthetic systems, and these consortia frequently result in higher biomass production relative to monocultures. The focus here is on the impact of initial spatial localization and substrate diffusivity on the growth of a model microbial consortium consisting of a producer strain that consumes glucose and produces acetate and a scavenger strain that consumes the acetate. The mathematical model is based on an individual cell model where growth is described by Monod kinetics, and substrate transport is described by a continuum-based, non-equilibrium reaction-diffusion model where convective transport is negligible (e.g., in a biofilm). The first set of results focus on a single producer cell at the center of the domain and surrounded by an initial population of scavenger cells. The impact of the initial population density and substrate diffusivity is examined. A transition is observed from the highest initial density resulting in the greatest cell growth to cell growth being independent of initial density. A high initial density minimizes diffusive transport time and is typically expected to result in the highest growth, but this expected behavior is not predicted in environments with lower diffusivity or larger length scales. When the producer cells are placed on the bottom of the domain with the scavenger cells above in a layered biofilm arrangement, a similar critical transition is observed. For the highest diffusivity values examined, a thin, dense initial scavenger layer is optimal for cell growth. However, for smaller diffusivity values, a thicker, less dense initial scavenger layer provides maximal growth. The overall conclusion is that high density clustering of members of a food chain is optimal under most common transport conditions, but under some slow transport conditions, high density clustering may not be optimal for microbial growth.

## Introduction

Microbes in nature are almost exclusively organized as consortia; including diverse microbial communities in the soil, ocean, and the human gut [1, 2]. Natural consortia often form syntrophic communities where the microbes depend on each other for the production of required metabolic substrates and/or the maintenance of chemically advantageous conditions [3–5]. Syntrophic cooperation often leads to an increase in productivity and can lead to advanced functions [6–8].

Early theoretical models of microbial growth led to the development of the competitive exclusion principle, which states that the maximum number of species that can coexist in a system is equal to the total number of limiting resources [9]. The existence of natural, stable microbial consortia is explained in a number of different ways, including: spatial heterogeneity or segregation, environmental fluctuations preventing equilibrium, and inter- and intra-species interactions [10].

The system of interest here is a cross-feeding chain where multiple microbes sequentially degrade a single substrate. Cross-feeding chains are common in natural systems including the degradation of lignocellulosic material [5, 11]. Another cross-feeding chain that has been found to evolve repeatedly in different experiments occurs when *E*. *coli* is grown using glucose as the substrate. After many generations of growth, the original *E*. *coli* strain splits into unique sub-strains: one strain consumes glucose and produces acetate, and another consumes acetate and oxygen [12, 13]. This cross-feeding template has previously been studied experimentally in a well-mixed, chemostat environment, and the cross-feeding chain consortia was found to be more productive than the original single strain of *E*. *coli* [6]. Note that productivity was defined as total biomass production per input of glucose. A cross-feeding *E*. *coli* chain has also been studied theoretically, and one possible explanation for the increased biomass production is a change in pathway efficiency [14] while another explanation is an improved yield based on regulation changes.

The spatial localization of the various microbes in a syntrophic system is not relevant when studying systems that are well-mixed, e.g., chemostats. However, spatial localization can be critically important for some systems where transport processes are limited [15–17]. In particular, microbial biofilms significantly limit convective mixing and the primary mode of substrate transport is via diffusion with biofilms [18, 19]. Spatial localization can have important implications for biofilms that contain a microbial consortia [20, 21]. For example, spatial localization has been shown to be significant for microbes in a granular sludge system for degrading terephthalate [22]. The microbes involved in periodontal diseases have also been shown to be spatially localized using digital image analysis [23]. The functionalities of the various microbial consortia in the gut microbiota has been shown to vary significantly with spatial location [24]. Finally, Bernstein et al. showed enhanced biomass productivity for a synthetic microbial consortia in a biofilm with spatial localization [6].

Recent results on the analysis of the role of spatial localization for collective enzyme activity has renewed interest in spatial localization of microbial consortia [25, 26]. From a mathematical standpoint, the chain of two enzymatically controlled reactions (i.e., *substrate* → *intermediate* → *product*) examined by Buchner *et al*. is similar to the syntrophic microbial chain examined here. Buchner *et al*. examined the spatial localization of the two enzymes in the chain and found that the system undergoes a transition from co-localization optimality (i.e., clustering or co-localization results in the highest flux from substrate to product) to an extended profile with pure clustering or co-localization not providing the highest flux. Because Buchner *et al*. studied a chain of two enzymatically controlled reactions, a very high density of enzymes was predicted to be optimal in many cases. The enzyme density was only limited in a few cases by Buchner *et al*. There is a limit to the density of enzyme or cellular systems because two enzymes or two cells cannot occupy the same volume, but this limit is much stricter for cellular systems than enzyme systems because of the larger length scales in cellular systems. The result of Buchner *et al*. on enzyme systems, however, motivated some of the question behind the work described here: how does spatial localization (i.e., clustering versus disperse spreading of microbes) impact productivity (i.e., total biomass change) for a simple cross-feeding chain consisting of two microbes?

Motivated by the work of Buchner *et al*., the first system examined here consists of a single producer cell that converts glucose into acetate at a constant flux and is surrounded by an initial population of scavenger cells that convert the acetate (and oxygen) into carbon dioxide. We examine the impact of the spatial density of the initial population and the diffusive transport rate of the substrate on the overall growth of the initial population of scavenger cells. Later, based on the experimental measurements of Bernstein *et al*., we examine the impact of initial spatial density and substrate diffusivity on overall growth in a biofilm system. In both systems, we observe a critical transition from a state in which clustering or high initial density is most productive to a state in which a less dense initial spatial arrangement is equally productive or more productive. A significant amount of prior research has focused on competition in microbial consortia, and most of this has focused on competition between different species and strains, e.g., [27–29]. Further significant work on individual or agent based biofilm models has also been completed, including multispecies biofilms and a few studies on spatial segregation in biofilms, e.g., [30–33]. The unique contribution of this work is that it is the first to examine the impact of initial density on the growth of the scavenger organism in a syntrophic chain using an individual based biofilm model and the first to observe the critical transition away from dense clustering being optimal. These results could have implications for the optimal density of both natural and artificial microbial consortia in biofilms.

## Methods

**Fig 1:**
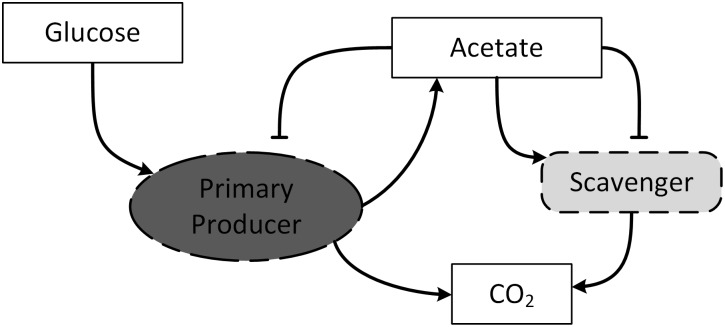
The two-species syntrophic chain with the primary acetate producer and acetate scavenger. Both species are inhibited by acetate.

The effects of spatial localization on the growth of a microbial consortia is investigated through the simplest case of a syntrophic chain of only two microbial species (Fig 1). The ‘primary producer’ microbe takes glucose (*G*) and produces acetate (*A*), and the microbe is also inhibited by high concentrations of acetate. The second microbe, the ‘scavenger’ consumes acetate and oxygen and produces carbon dioxide. The second microbe, like the first, is also inhibited by acetate at high concentrations. This model consortia is used here due to its simplicity, previous analysis in environments without spatial localization [14], and the availability of experimental measurements [6].

### Individual Based Biofilm Model

Biofilms are aggregates of cells within a polymeric matrix of microbial origin that form on many different surfaces after microbial attachment [34, 35]. Many different mathematical models have been proposed for describing various properties of the biofilm, including cell growth, biofilm detachment, substrate diffusion, and chemical reaction kinetics [36]. Biofilms contain individual cells, extracellular polymeric substances, and a number of different substrates that are transported within the biofilm, primarily by diffusion [19, 37]. Some of the substrates are utilized as a food or energy source by the cells while other substrates may be waste products from the cells or inhibitors/toxins that reduce cell growth and viability. The unique environment of the biofilm—individual cells interacting with diffusible substrates in a confining network—has led to the development of a number of individual based (or agent based) biofilm models [30, 32, 33, 38, 39]. These models typically contain individual cells that are modeled as discrete entities with defined metabolic and growth properties, and they contain one or more diffusible substrates that are modelled using a continuum approximation. The two parts of the individual based biofilm model—the microbial cell model and the diffusible substrate model—are described first, and then the overall algorithm for combining the cells and substrates into a biofilm model is described later.

### Microbial Cell Model

Individual based biofilm models can theoretically include a large number of different microbial species within a single biofilm. Here, only two microbial species are considered. The first species, called the ‘producer’ converts glucose into acetate and its growth rate can be described by:

$$\frac{dW}{dt}=\left(\frac{\mu_{W}\cdot G}{K_{G}+G}\right)\left(\frac{K_{IW}}{K_{\text{IW}}+A}\right)W$$ (1)

where *G* is the local glucose concentration (g/L), *μ*_*W*_ is the maximum growth rate constant, *K*_*i*_ is the half-saturation concentration for substrate *i*, and *K*_*IW*_ is the acetate inhibition constant for the primary producer. To simplify the analysis of spatial location effects, the producer species is frequently assumed to convert glucose to acetate at a fixed, maximum rate (2.2 pg/h), the cells do not grow or divide, and they are not effected by acetate inhibition for the results shown here unless stated otherwise. The second species, called the ‘scavenger’, consumes acetate and each cell grows at a rate:

$$\frac{dX}{dt}=\left(\frac{\mu_{A}\cdot A}{K_{A}+A+\frac{A^{2}}{K_{IX}}}\right)\left(\frac{O_{2}}{K_{O_{2}}+O_{2}}\right)X$$ (2)

where *A* is the local acetate concentration (g/L), *μ*_*A*_ is the maximum growth rate constant, and *K*_*IX*_ is the acetate inhibition constant for the scavenger cells. The parameters in the cell growth model were based on various experimental measurements and are consistent with those used previously in model of the same microbial consortium (see [14] and the references therein). The parameters used in the basic model are listed in Table 1.

**Table 1:** Parameters used in the baseline version model. See [14] and references therein for source information.

Parameter	Value	Parameter	Value
***μ***_***W***_	0.7 h^-1^	***μ***_***A***_	0.3 h^-1^
***K***_***G***_	0.05 g/L	***K***_***A***_	0.05 g/L
***K***_***IW***_	0.4 g/L	***K***_***IX***_	0.7 g/L
***Y***_***GW***_	0.15 mg cdw / mg G	$K_{O_{2}}$	2.75x10^-4^ g/L
$D_{Glu}$	0.85 mm^2^/h	***Y***_***AX***_	0.40 mg cdw / mg A
		$D_{A}$	0.994 mm^2^/h
		$D_{O_{2}}$	8.9 mm^2^/h

The scavenger growth rate, $\frac{dX}{dt}$, is calculated at each discrete time step of the biofilm simulation, and the mass of the cell is increased based on the growth rate. As the mass of the cell increases, the radius of the cell is adjusted based on a constant density assumption. The initial cell mass is set to 0.4 pg, and the radius is assume to be approximately 1 *μm* for determining the spatial volume occupied by the cell. Once the mass of a cell has exceeded a preset threshold (a default threshold mass of twice the initial mass is used here) the cell divides into two cells, and the new cell is assigned a random location approximately adjacent to the original parent cell.

### Diffusible Substrate Model

The kinetics and transport of multiple substrates within the biofilm are modeled using a reaction-diffusion equation [40] of each individual substrate, *S*:

$$\frac{dS}{dt}=D_{S}\nabla^{2}S-\frac{1}{Y_{si}}{\left(\frac{dX}{dt}\right)}_{i}$$ (3)

where $D_{S}$ is the diffusivity of substrate *S* in the biofilm, *Y*_*si*_ is the yield of species *i* growing on substrate *S*, and ${\left(\frac{dX}{dt}\right)}_{i}$ is the growth rate of cell *i*. The approximate solution to Eq (3) is obtained using a second-order finite difference approximation for spatial derivatives and mesh using with at least 4000 nodes (i.e., 64 nodes by 64 nodes). The temporal derivative was discretized using either a second-order implicit time stepping algorithm (Crank-Nicolson) or a first-order implicit time stepping algorithm (backward Euler) for the temporal derivative [41]. The time step was set sufficiently small (Δ*t* = 3 × 10^−4^ *h*) so that the approximate solutions differed by less than 0.1% between the first-order and second-order implicit time stepping algorithms. The growth term is based on discrete, individual cells in the domain so the kinetic term in Eq (3) is enforced on the finite difference points surrounding the cell using linear interpolation. Specifically, the substrate concentration and corresponding cell growth rate for the individual cell at a specific location is determined by linearly interpolating the substrate concentration from the 4 surrounding finite difference nodes to the cell location. Then, based on the calculated growth rate, the resulting substrate concentrations at the surrounding finite difference nodes are adjusted based on the volume of fluid associated with the finite difference node. Overall, the approach is very similar to that used in previous individual-based microbial models.

The primary focus here is on the impact of spatial localization on cell growth for the scavenger species in a syntrophic chain. The only spatial derivative in the above equations is the diffusion term in the substrate equation, so the fundamental question is the relationship between spatial diffusion and substrate conversion into biomass. In order to focus on this specific interaction, the diffusivity, D, will be the primary model parameter that is varied for the different systems shown below, and the other model parameters that all relate to substrate kinetics (*Y*_*si*_, *μ*, *K*_*I*_, $K_{o_{2}}$, *K*_*A*_) will be set to a fixed value, estimated from published experimental measurements (Table 1), unless otherwise noted. In natural, bacterial food chains in a biofilm, a number of factors can impact the diffusivity of substrates including the composition and density of the biofilm, temperature, and size/molecular weight of the substrate. There is an approximately linear relationship between temperature and diffusivity and there is an inverse relationship between the square root of molecular weight and diffusivity [42].

### Combined Biofilm Model

The individual based biofilm model used for the spatial localization analysis below was developed using the Python programming language and is implemented using an object oriented programming model (source code publically available at https://github.com/jeffheys/biofilmSegregation and Montana State University ScholarWorks, http://doi.org/10.5072/FK2M907F0G). The models are all 2-dimensional and the domain is a 0.2 mm by 0.2 mm square unless otherwise stated. Simulations begin by creating and locating an initial population of producer and scavenger cells with the locations of the scavenger cells always being random locations within a specified region. Next, a regular mesh of nodes (typically 64 x 64 nodes) is defined over the domain and an initial concentration and boundary conditions are defined for each substrate. The initial concentration within the domain is set to zero for all substrates (acetate, glucose, and oxygen). The default boundary condition is a zero flux (or wall) boundary condition for each substrate on the left, right, and bottom walls, and a Dirichlet boundary condition is set on the top boundary to represent the surface of a biofilm open to the environment. For acetate, the concentration is set to zero along the top surface, and for oxygen, the concentration is set to 0.008 g/L based on air saturation. The bulk glucose concentration on the top surface is set to 1.0 g/L.

After the initial setup of the cells and substrates, a time loop is used to simulate the growth of cells and substrate transport for the desired time period. After each time step, the growth of each individual cell is calculated and the cell mass is increased. Based on the growth rate of each cell, substrate concentrations are adjusted—substrates are either consumed or produced using the yield value for the cell—at each finite difference node immediately surrounding the cell. Any cells that grow beyond a predetermined threshold (the default value used here is twice the original cell mass) are divided into two cells, which are reset to the original cell mass. Of the two cells that result from cell division, one cell retains the location of the original cell and the other cell is placed at a random location within one-and-a-half cell diameters of the original cell location. After the growth and possible division of the individual cells, a spreading process is used to check for overlap between cells and any overlap that is found is eliminated out by moving cells a small distance from one another. Multiple spreading iterations are often required to eliminate all instances of cell overlap, especially when cell numbers exceed 100 cells in the model domain.

## Results

**Fig 2:**
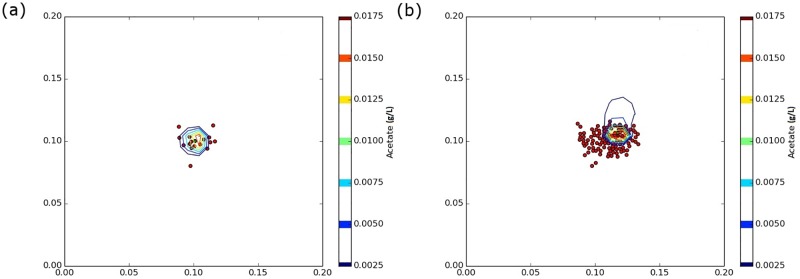
(a) An initial population of 16 scavenger cells (red) randomly located in a cluster of radius 0.02 mm around a primary producer (blue). (b) The scavenger cell cluster after 45 hrs of growth and contours showing acetate concentration in g/L.

The analysis of the effects of spatial segregation focuses on the impact of the initial spatial distribution of a population of scavenger cells on future cell growth. Fig 2a shows an initial population of 16 scavenger cells in a circular cluster with a diameter of 0.04 mm surrounding a single producer cell that is at the center of the unit square domain. Using a diffusivity, D, of 0.994 mm^2^/h and allowing the initial population to grow for 45 hours results in cell growth, cell division, and spreading into the cell cluster shown in Fig 2b. The contours of acetate concentration, *A*, in g/L are also shown in Fig 2b. The highest concentration is normally surrounding the producer cell. Islands of low acetate concentration are sometimes present in the cell cluster where acetate has been depleted by an especially dense population of scavenger cells. The choice of starting with 16 scavenger cells is an arbitrary choice. Testing showed that using a smaller number of starting cells caused large stochastic differences between simulations due to the random initial cell locations, and 16 initial cells was a good balance between overly large and overly small stochastic differences between simulations.

**Fig 3:**
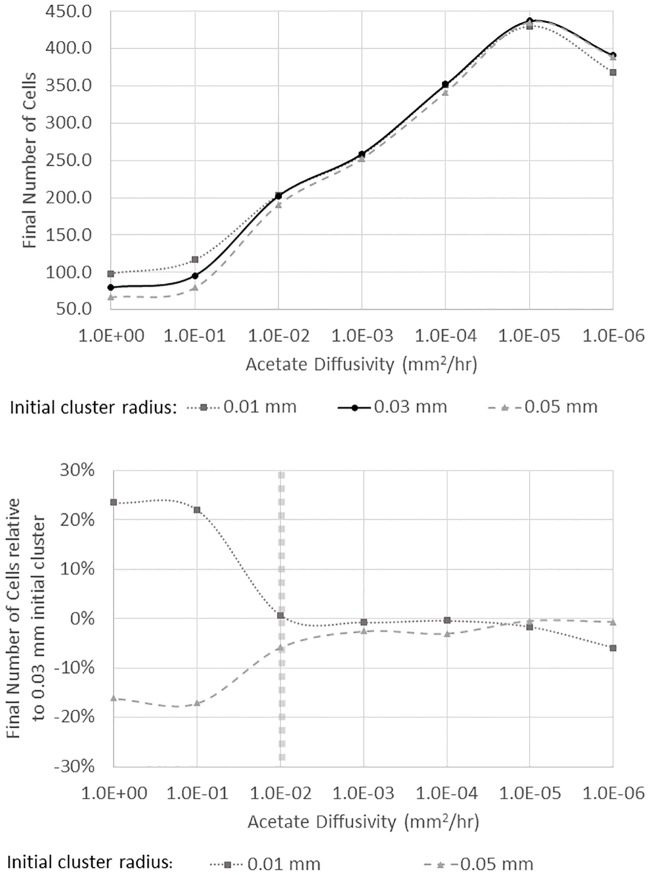
Final number of cells from an initial cluster of 16 scavenger cells for three different initial cluster sizes with different acetate diffusivity values (upper panel). The lower panel shows the same information but the final cell population is shown as a percentage of the medium density initial cluster that had 16 cells in a circle with a radius of 0.03 mm. The vertical, dashed line marks the critical transition between a higher initial density resulting in greater growth to a regime where total growth is largely independent of initial density. The results shown represent the average of 8 simulations, and error bars are not visible as they same size as the symbols).

The interplay between acetate diffusivity and the initial spatial segregation of the cells is examined in Fig 3. Acetate diffusivity is varied from 1.0 *mm*^2^ / *h* to 1 × 10^−6^ *mm*^2^ / *h* with the highest diffusivity being shown on the left and the lowest diffusivity being shown on the right side of Fig 3, consistent with [26]. The growth of the initial population of 16 scavenger cells is measured in terms of the total number of cells after 100 hours of growth and is shown on the y-axis. Larger values of diffusivity, shown on the left side of Fig 3, result in lower acetate concentrations and, hence, lower growth rates for the initial population of scavenger cells. When the initial cluster of scavenger cells is more densely packed near the producer cell (i.e., the initial cluster radius of 0.01 mm), a higher growth rate is observed than for the cases of the larger initial cluster of scavenger cells. This observation is expected because it is advantageous in this case to have as many cells as possible in the region of higher acetate concentrations. For diffusivity values of 1 × 10^−2^ *mm*^2^ / *h* or less, shown on the right side of Fig 3, the situation is less clear. The smaller diffusivity leads to higher acetate concentrations and higher growth rates for all initial cell cluster sizes, but it is no longer the case that the smallest and densest initial cluster has the largest final population. If the substrate diffusivity is less than 1 × 10^−2^ *mm*^2^ / *h*, the growth rate is largely independent of the density (or clustering of the initial population). Specifically, for substrate diffusivity values of 1 × 10^−3^ − 1 × 10^−5^ *mm*^2^ / *h* the final populations were not significantly different based on a t-test (p > 0.05). In the case of the smallest diffusivity values examined, 1 × 10^−6^ *mm*^2^ / *h*, the largest and least dense initial cluster of 16 scavenger cells has the largest final population.

Buchner et al. [25, 26] observed a transition from a state where clustering of enzymes was optimal at higher diffusivity to a state were clustering was not optimal at lower diffusivities. The result for enzymes was attributed to substrate that diffused away from the central cluster but was still captured by more distant enzymes. The case here is somewhat different because the larger length scales involved result in minimal escape of substrate, but the result here also shows a transition away from clustering being optimal for growth to a situation where overall growth is less dependent on initial cluster density. It is also important to note that the initial population of scavenger cells was placed in random locations, but for all conditions examined, the final population is based on an average of 8 simulations with different initial random cell locations. The standard deviation of the final population varied between ±5 cells at the lowest growth conditions to ±10 cells for the higher growth conditions.

The range of substrate diffusivity values examined in Fig 3 is from 1.0 *mm*^2^ / *h* to 1 × 10^−6^ *mm*^2^ / *h* and is clearly very large. Diffusivity values larger than 1.0 *mm*^2^ / *h* were modeled, but the results are not shown because the growth trends did not change significantly from those at 1.0 *mm*^2^ / *h* and systems with significantly higher diffusivities are not biologically realistic. Acetate diffusion in biofilm systems has an estimated diffusivity of slightly less than 1.0 *mm*^2^ / *h*, but microbial consortia that exchange a higher molecular weight substrate in a more restrictive diffusive environment (i.e., an animal or plant tissue) could have a substrate diffusivity on the order of 1 × 10^−2^ *mm*^2^ / *h*. In this regime, the impact of clustering or density differences for the initial population would be much less on overall growth than the higher diffusivity system. Clearly, the simulations at extremely low diffusivity values of 1 × 10^−6^ *mm*^2^ / *h* represent systems that are unlikely to be found in nature. However, it is important to stress that the impact of initial spatial segregation and clustering on overall growth changes significantly over a range of physically plausible diffusivity values (i.e., from 1 *mm*^2^ / *h* to 1 × 10^−2^ *mm*^2^ / *h*), and microbial consortia have been found in extreme environments including those with extremely low ‘effective’ diffusivities such as ice [43].

**Fig 4:**
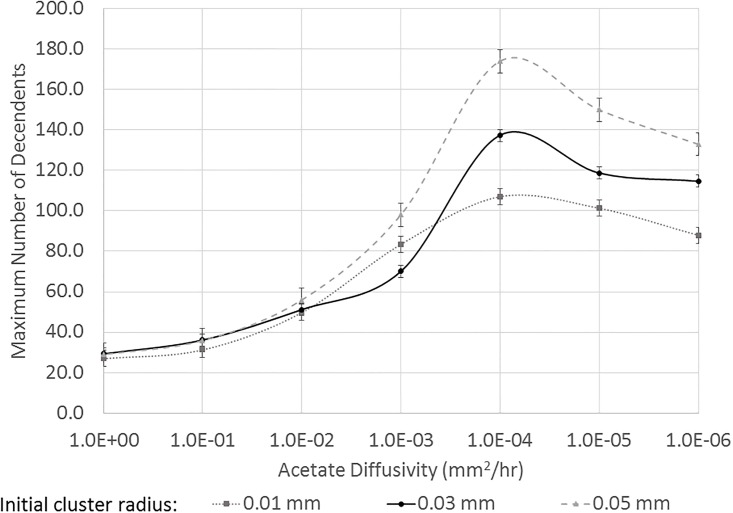
The maximum number of descendants from one of the 16 original scavenger cells. At high diffusivity values, the acetate concentration is more uniform and all cells reproduce at a similar rate. At low diffusivity values, a few cells that are located near the original acetate source reproduce more rapidly, especially for a cluster with a lower initial density.

To gain some insight into the result shown in Fig 3, it is useful to examine the number of descendants from a cell in the initial cluster of 16 scavenger cells. Specifically, the maximum number of descendants from a single cell is shown in Fig 4 for the different initial cluster sizes and acetate diffusivity values. For all the different conditions tested, including different diffusivity values and different initial cell densities, one (or a few) initial scavenger cells that are initially closest to the producer cell are responsible for the vast majority of the decedents. At high diffusivity values (left edge of Fig 3), the acetate concentration is more uniform across the domain and it is advantageous to have as many initial cells as possible near the food source. The very closest cells have the most descendants, but the other nearby cells have some descendants. For lower diffusivity values, which lead to higher local acetate concentrations near the producer cells, the single closest initial cell results in the majority of the descendants. The larger initial cluster (initial cluster radius of 0.05 mm) results in more food being available to the most prolific cell(s) near the food source and having a few cells further away from the producer that capture and eat the acetate before it leaves the domain results in the largest initial cluster having similar (or slightly greater) overall growth than the denser initial clusters despite the larger initial clusters have half or fewer cells close to the cluster initially. This result is also similar to that observed by Buchner et al. [26], but the physical phenomena here are somewhat different due to the larger length scales.

The propagation success of the various scavenger cells within the domain is dominated by the individuals that initially occupy locations nearest the producer cell (i.e., nearest the food source). As the closest scavenger cell(s) grow and divide, their progeny push away other scavenger cells that individually occupied locations slightly further from the producer cell and away from the food source. In this way, the very best reproductive strategy for the individual is to occupy locations as close as possible to the food source. Chemotaxis, which is not modeled here, could be beneficial for maximizing individual reproduction. However, the best strategy for a group of scavenger cells is not always to maximize clustering near the food source. In many of the random simulations at lower diffusivity conditions, having some scavenger cells further away from the food source was the optimal strategy for the group to maximize the collective number of descendants. In other words, what is best for the individual (clustering) is not always best for the community.

**Fig 5:**
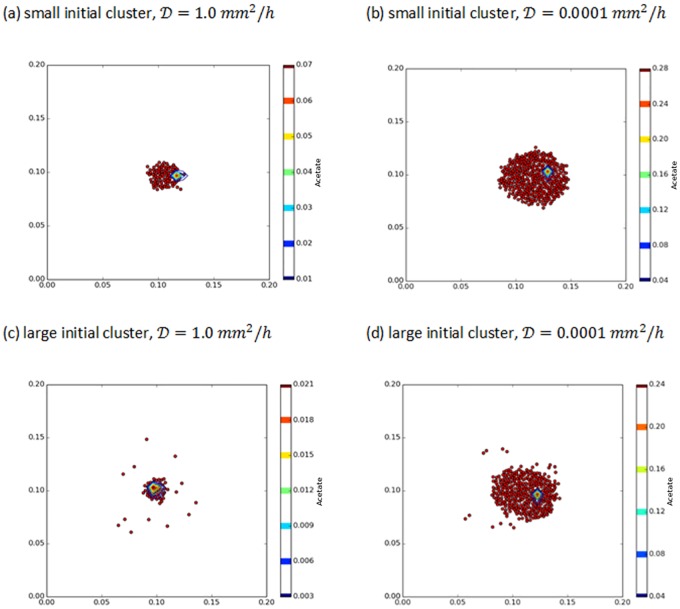
Final populations of scavenger cells (red) and the single producer cell (blue) for different initial cluster sizes and acetate diffusivity values. All acetate concentrations are in g/L.

Additional insight into the impact of initial cluster radius on scavenger cell growth at low diffusivity is shown in Fig 5. A small initial cluster, Fig 5(b), results in robust growth, but the dense population of scavenger cells initially reduces the acetate concentration relative to the larger initial cluster shown in Fig 5(d). The impact of the initial cluster size is not dramatic, but it is sufficient to give the slightly larger, less dense initial cluster an equal rate of overall cell growth—especially for the few scavenger cells initially located near the producer cell. These cells have a much larger number of descendants compared with the higher density (and lower acetate concentration) and smaller initial clusters. This result is consistent with the model predictions of Buchner et al. [25, 26] for a similar spatial localization problem with enzymes.

**Fig 6:**
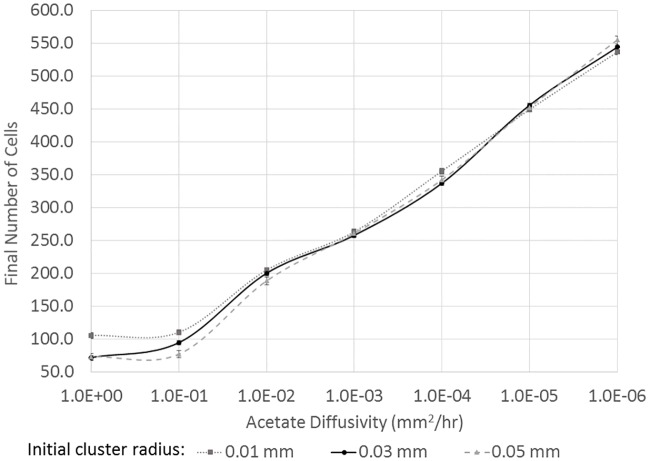
Final number of cells from an initial cluster of 16 scavenger cells for a system without acetate inhibition. Three different initial cluster sizes were modeled with different acetate diffusivity values. Final number of cells represents the average of 8 simulations and the final populations at diffusivity values of 1.0 × 10^−3^ *mm*^2^ / *h* and 1.0 × 10^−5^ *mm*^2^ / *h* were not statistically different at 95% confidence based on a t-test.

The growth model for the scavenger cells includes an inhibition term where the growth rate is reduced at high concentrations of acetate. Growth inhibition due to the high concentration of a single substrate, in this case the waste product of the producer cell, is extremely common and is probably more accurate than ignoring inhibition. However, to test the robustness of the results shown in Fig 3, acetate inhibition for the scavenger was removed from the growth rate model (Eq 2), and the impact of initial cluster size and acetate diffusivity of overall growth was again assessed. These results are shown in Fig 6. For the highest diffusivity values examine (left side of Fig 6), the results are similar to those with inhibition: the smallest initial cluster had the largest overall cell growth and the less dense, larger clusters had lower cell growth due to lower acetate concentrations. At moderate diffusivity values (1.0 × 10^−2^ *mm*^2^ / *h*), the effects of initial cluster size vanished, which is also consistent with the previous results with inhibition. At the smallest diffusivity values (right side of Fig 6), higher cell growth is observed due to higher acetate concentrations, and the lack of inhibition in this case leads to larger final cell counts than shown in Fig 3.

The final cell count as a function of diffusivity for the system with inhibition shown in Fig 3 has a maximum at $D=1\times 10^{-5}\;mm^{2}/h$ regardless of the initial cluster size. As the diffusivity decreased from 1.0 *mm*^2^ / *h* to smaller values, more acetate persisted near the producer cell and near the cluster of scavenger cells for a longer period of time and less acetate escaped out the top boundary. Thus, lowering the diffusivity led to an increase in scavenger cell growth until it reached a maximum at $D=1\times 10^{-5}\;mm^{2}/h$. At even lower diffusivity values than this, the acetate moved outward from the producer cell so slowly that it reduced scavenger cell growth because acetate concentrations were so low only a short distance away from the producer. The final cell count as a function of acetate diffusivity for the system without inhibition (Fig 6) does not show a maximum at the same acetate diffusivity values ($D=1\times 10^{-5}\;mm^{2}/h$). For this system, the maximum growth rate occurs at a diffusivity value of approximately 5 × 10^−7^ *mm*^2^ / *h*, but this value is a rough estimate due to discretization errors in solving the acetate transport equation with such a small diffusivity value. The very small diffusivity values result in more acetate remaining close to the producer cell and less acetate escaping the domain. The higher concentration near the producer cell also potentially results in more inhibition near the producer cell. Thus, the system with inhibition has a larger diffusivity leading to maximum cell growth than the system without inhibition.

### Domain Scaling

As described previously, the diffusivity of common microbial substrates in biofilms and other hydrated environments does not vary over the range of values that is examined here. There will clearly be some variability due to the substrate and there will be even greater variability between heavily hydrated environments, such as a biofilm, and a denser environment such as a tissue or soil. However, even by examining a wide range of substrate molecules, variations in temperature, and variations in the permeability of the environment, the diffusivity values would only vary by approximately two orders of magnitude and less than the 5 or 6 orders of magnitude examined above. However, the spatial segregation effects examined above can arise from changes other than just a change in diffusivity. The substrate transport equation (Eq 3) can be non-dimensionalized by defining a characteristic length scale, *L*, a characteristic time scale for microbial growth, *T*, and a characteristic substrate concentration, *S*_0_. The non-dimensionalized form of Eq 3 can then be written:

$$\frac{d\hat{S}}{d\hat{t}}=\left(\frac{D\cdot T}{L^{2}}\right){\hat{\nabla }}^{2}\hat{S}+\frac{1}{Y_{si}S_{0}}{\left(\frac{dX}{d\hat{t}}\right)}_{i}$$

where $\hat{S}=S/S_{0}$, $\hat{t}=t/T$, and $\hat{\text{x}}=\text{x}/L$. From the perspective of diffusive substrate transport alone, there is equivalence between varying the diffusivity and varying the size of the domain, *L*.

Instead of simply varying the diffusivity, D, the size of the domain, *L*, can be varied over only 2 or 3 orders of magnitude to get a system that in some ways is mathematically similar to varying the diffusivity by 4 or 6 orders of magnitude. However, substrate transport is just one of the physical phenomena present in the system, and other phenomena such as substrate production and cell growth will be impacted differently through domain size changes. For example, increasing in the model domain from 0.2 mm by 0.2 mm to a domain that is 20 mm by 20 mm causes a significant decrease in the average amount of substrate available to the scavenger due to the much larger area available for transport. Partial compensation for this is possible by extending the simulation time to a few 1000 hours or 10’s of thousands of hours, but that is a situation that is unlikely to exist because few systems are stable and unchanging for that length of time. A more plausible alternative is to imagine a substrate source with a greater production rate. For example, the source could be many producer cells or a non-biological source. The source is still modeled as a single point for consistency with previous simulations, but the productivity of the source is increased as the length-scale is increased (specifically, substrate production is scaled as: ${\dot{S}}_{large}={\dot{S}}_{small}\cdot {\left(\frac{L_{large}}{L_{small}}\right)}^{1.5}$ where $\dot{S}$ is the substrate production rate and *L* is the length of one edge of the square domain). The exponent, 1.5, was chosen arbitrarily, but the value needed to be large enough that the quantity of acetate produced increased as the area increased, but the value could not be too large or the production and growth became unrealistic.

**Fig 7:**
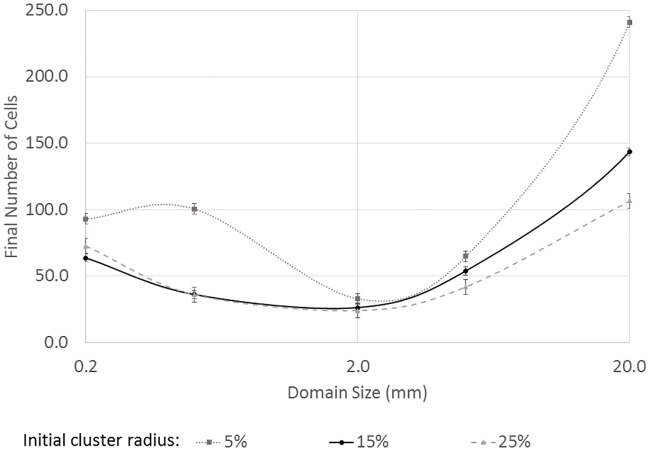
The effects of domain size and initial cluster size on the overall growth using a fixed diffusivity for the substrate. As the domain size increases, there are competing effects on growth of substrate dilution and increased substrate production at the center of the domain by the producer cell.

The results of increasing the domain size (and acetate production) are shown in Fig 7. The radius of the initial cluster of 16 scavenger cells was set to a specific fraction of full domain size from 5% of the full domain to 25% of the full domain, consistent with previous simulations. For the smallest domain, which is the same size used for all previous simulations (0.2 mm by 0.2 mm), it can be seen that the smallest initial cluster leads to the greatest growth. This is consistent to what was observed earlier for the highest substrate diffusivities simulated. As the domain increases in size, it is mathematically related to a decrease in the diffusivity, and as before, the smallest initial cluster becomes less dominant and a larger initial cluster is observed to have equal growth for a 2 mm by 2 mm domain. For even larger domains (i.e., larger than 2 mm), the increase in acetate production causes high concentrations in the area around the producers, in some cases the concentration is sufficient to cause some inhibition, but the smaller initial clusters with more cells initially near the producer still have the highest growth rate.

**Fig 8:**
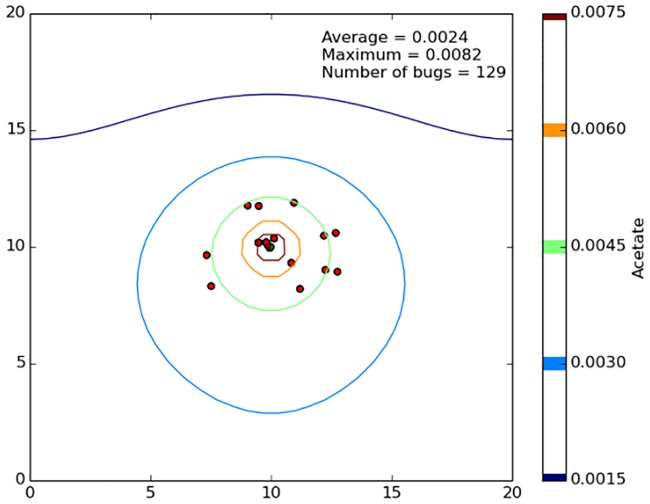
Final population when starting with 16 scavengers randomly located with a circle that was 30% of the domain diameter, but the domain is a significantly (100x) larger than before. The final population has 129 cells in a few dense clusters.

For the largest domain sizes, i.e., the right side of Fig 7, the cells were very small relative to the size of the domain, and the growing cells formed small clusters relative to the size of the domain (Fig 8). For a diffusing substrate molecule, these small clusters are less likely to be encountered when they are spread out spatially further from the producer cell. Hence, at the larger length scales there is less benefit to a larger initial cluster.

### Layer Initial Populations

Biofilms that contain a mixture of microbes are often observed to have the microbes separated into distinct layers. These biofilms are commonly referred to as ‘microbial mats’ and they often contain a syntrophic chain of microbes. To examine the impact of spatial segregation and diffusion rates on the growth of a microbial mat, the initial state of the model syntrophic chain was modified to contain 32 producer cells randomly located very near (i.e., within the bottom 1%) the bottom of the domain. Once again, the producer cells were limited to a fixed production rate of acetate and they were prevented from growing or dividing. Then, 32 initial scavenger cells were placed in the domain in a layer of variable thickness near the bottom of the domain. The thickness of the scavenger layer varied between 2.5% of the total domain thickness and 20% of the total domain thickness. The diffusivity of acetate was then varied over a number of orders of magnitude to better assess the impact of the initial scavenger layer thickness and acetate diffusivity on growth.

**Fig 9:**
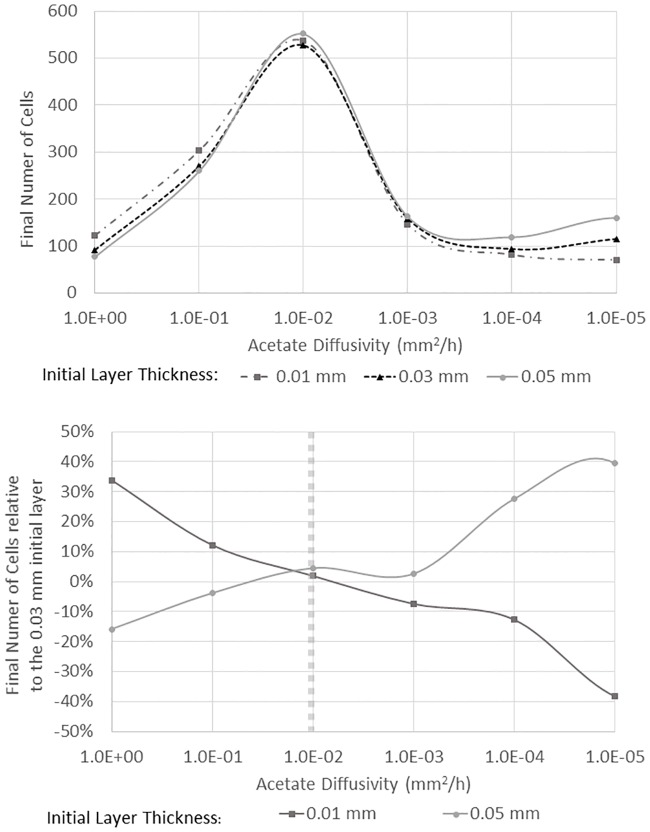
Impact of the thickness of the initial layer of 32 scavenger cells and acetate diffusivity on total cell growth for a biofilm (top). The domain included 32 producer cells near the bottom boundary. The lower panel shows the same information but the final cell population is shown as a percentage of the medium initial layer thickness (0.03 mm). The vertical, dashed line marks the critical transition between the region where a higher initial density (i.e., thinner initial layer) resulting in greater growth to a regime where a less dense initial layer resulted in the greatest growth.

**Fig 10:**
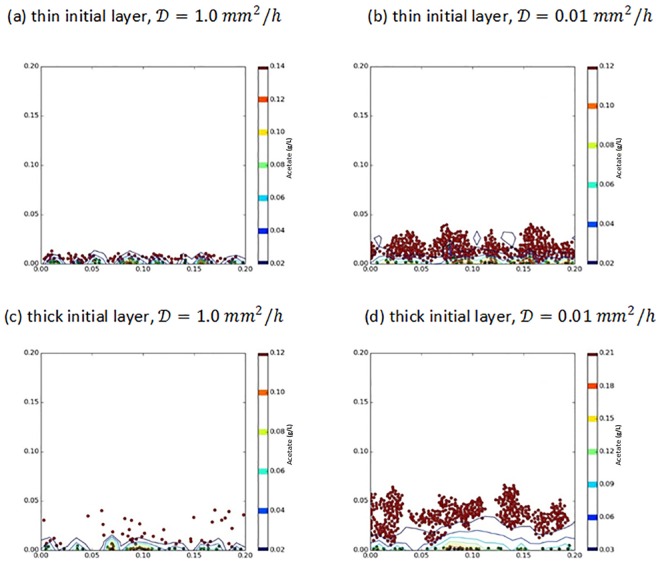
Final microbe locations for a ‘thick’ initial layer and ‘thin’ initial layer for two different acetate diffusivity values.

Growth was quantified by counting the total number of individual cells in the domain (averaged over 8 independent simulations) after a fixed time period (20 hours) as shown in Fig 9. For the case of a layered biofilm, the initial spatial segregation, i.e., the thickness of the initial scavenger layer, had an even greater impact on overall growth than in the situation of a single producer cell surrounded by scavenger cells. In the previous case, there was a transition from a small, high density initial cluster being optimal at the largest diffusivities examined to a regime in which overall cell growth was largely independent of the initial cluster size or density. For the layered case, there was also a transition, but in this case the transition was even stronger. For the highest diffusivity values examined, which are shown on the left side of Fig 9, a thinner, denser initial cluster led to larger growth rates. For these high diffusivities, the overall acetate concentration was low and overall growth rates were low, so it was an advantage for the scavenger to be as close as possible to the acetate source—the producer cells—along the bottom edge. At moderate acetate diffusivity values, i.e., $D=0.01\;mm^{2}/hr$, the overall growth was larger for all initial layer thicknesses, but the thickest initial layer showed the highest growth rates. As shown below in Fig 10, the moderate diffusivity values favored a less dense initial scavenger layer and resulted in the highest overall growth rates that were observed. At very small acetate diffusivity levels, the very low diffusivity values limited growth, but the thicker initial layer of individual scavenger cells continued to show the largest overall cell growth. For all diffusivity values shown, at least one final scavenger population was statistical different at 95% confidence from one of the other final populations at the same diffusivity.

For the layer biofilm system, simulations were also run where the primary producer cells where allowed to grow and multiply just like the scavenger cells. This change makes it difficult to assess the impact of spatial segregation on a microbial food chain because both microbe populations are now changing and all the individual microbes moving instead of just one strain, the scavenger cells, as was done previously. In order to quantify growth in the biofilm system with both species growing and dividing, two different scenarios were compared. In the first scenario, the initial population of producer cells were randomly placed near the bottom on the left half of the domain and the initial scavenger cells were randomly located on the right half of the domain. This scenario is referred to as the ‘segregated’ scenario because the initial population of cells is segregated within the domain. For the second scenario, the initial population of cells are evenly spaced along the bottom of the domain with the producer cells and scavenger cells alternating in the sequence. This scenario is referred to as ‘interlaced’ because the initial population is interlaced with ever cell’s neighbors being the other member of the food chain. Table 2 compares the final population for the two different scenarios. The interlaced scenario leads to a 14% increase over the segregated scenario for the final population due to the reduced diffusion distances and slightly lower inhibition. To quantify the impact of acetate inhibition on overall growth, the simulations were repeated with the acetate inhibition effects doubled. The final population is reported in Table 2 and shows a slightly lower final population due to increased inhibition and a greater difference in final population between the segregated and interlaced scenarios.

**Table 2:** Final average population (based on 8 simulations) for a biofilm simulation with both producer and scavenger cells dividing. The segregated scenario has the initial populations of producer and scavenger separated on individual halves of the domain. The interlaced scenario has an initial population where the producers and scavenger cells alternate along the bottom of the domain.

	Segregated	Interlaced
**Normal**	233	266
**Acetate inhibition doubled**	191	256

## Conclusions

When considering a food chain, it is natural to assume that minimizing spatial separation and, thus, minimizing transportation costs, is an optimal strategy for conserving energy and food and maximizing growth. The results described here show a more complex situation. For the first geometry considered here—a single producer cell surrounded by multiple scavenger cells—the natural assumption that the system should minimize distance to the scavenger cells by maximizing the density of the scavenger cells is shown to be correct for higher substrate diffusivity values. However, a transition is observed as substrate diffusivity is decreased, and it is shown that some spatial separation and lower initial density for the scavenger leads to a similar (and in some cases greater) growth to the highly clustered case. For lower diffusivity values, a few cells near the producer divide prolifically. It appears to be beneficial to avoid very high densities of scavenger cells in order to allow higher overall substrate concentrations. This result still held even when substrate inhibition was not present, and, to some extent, it also held when the size of the spatial domain was increased instead of the diffusivity changing. The second geometry that was considered mimicked a biofilm with a thin layer of producer cells along the bottom of the domain and a second layer of scavenger cells above the producer cells. In this case, more distinct transitions were observed. At high diffusivity values, a thin, dense initial layer of scavenger cells resulted in the greatest amount of cell growth. At low diffusivity values, however, a thicker, less dense initial layer of scavenger cells leads to the greatest growth. These results demonstrate that the common assumption of minimizing transport costs and spatial separation may be an over simplification of the impact of spatial localization, and, under some conditions, some initial spatial separation may not impact overall population growth.

The limitations of this study include a fixed producer population that, in most cases, produced acetate at a fixed rate. Also, in the actual biological system upon which this model is based, the producer is inhibited by acetate and there is competition between the producer and scavenger for oxygen. The real biological system is 3-dimensional and usually lacks the artificial boundaries that were used in the model. Finally, biological systems and especially biofilms are inherently noisy, variable environments with random differences between cells and spatially heterogeneous transport rates.

This study was motivated by the work of Buchner et al. on the effects of spatial localization on a chain of two enzymatically controlled reactions. The impact of changing the diffusivity on overall microbial growth is qualitative similar to what was observed by Buchner even though the two systems have obvious differences. Finally, the results here show that as research on microbial consortia continues in areas such as gut microbiota, dental plaques, and waste water treatment, it could be important to consider the transport rates for substrates in these systems. The results in this paper show that under conditions of higher transport rates, it could be very beneficial to obtain a structure where the various members of a microbial food chain are in very close initial proximity to maximize the overall productivity of the system. However, under conditions where transport rates are highly limited for some reason, the initial configuration of the various members of a food chain may be less critical than one might otherwise expect. As long as a few members of each group in the food chain maintain a close initial proximity, that is likely sufficient for maximizing the overall productivity of the system.
